# Strengthening Community Health Worker and *Promotora* Workforce Capacity

**DOI:** 10.1001/jamanetworkopen.2026.6037

**Published:** 2026-04-10

**Authors:** Patricia Rodriguez Espinosa, Yessica Martinez Mulet, Nicole Wolfe, Nancy J. Burke, Dara H. Sorkin, Robynn Zender, Kristen L. Weeks-Norton, Gerardo Balcazar, Claudia G. Corchado, Sergio Aguilar-Gaxiola

**Affiliations:** 1 Department of Epidemiology and Population Health, Stanford School of Medicine, Palo Alto, California; 2 Office of Community Engagement, Stanford School of Medicine, Palo Alto, California; 3 Clinical and Translational Science Institute, University of Southern California, Los Angeles; 4 Department of Public Health, University of California, Merced; 5 University of California, Irvine; 6 Center for Reducing Health Disparities, University of California, Davis School of Medicine; 7 Visión y Compromiso, Los Angeles, California; 8 Cultiva Central Valley, Merced County, California; 9 Department of Internal Medicine, University of California, Davis School of Medicine; 10 Clinical and Translational Science Center, University of California, Davis School of Medicine

## Abstract

**Importance:**

Community health workers and *promotores* (CHW/Ps) are a crucial workforce supporting health promotion, resource navigation, and dissemination of evidence-based health information across diverse communities.

**Objective:**

To describe the process and best practices for codesigning and coimplementing innovative, scalable, and culturally relevant capacity-building trainings for CHW/Ps.

**Design, Setting, and Participants:**

This mixed-methods study employed a quantitative and qualitative approach and an implementation process involving a collaborative approach that integrated feedback from CHW/Ps, community partners, and advisory board members to select relevant workshop topics. Trainings were attended by CHW/Ps from 243 zip codes across California between September 2023 and March 2024.

**Exposure:**

CHW/P participation in culturally and linguistically relevant training workshops delivered monthly over videoconference. Workshops fostered engagement through interactive formats, multilingual resources, and accessible online platforms.

**Main Outcomes and Measures:**

Using data from a California-wide partnership, mixed methods (preworkshop and postworkshop sureys analyzed with paired sample *t* tests, qualitative data analyzed with thematic analysis, ripple effects mapping sessions, and listening sessions with attendees) were used to assess the feasibility, acceptability, and effectiveness of the CHW/P trainings.

**Results:**

A total of 505 CHW/Ps (469 female [93%]; mean [SD] age, 47.1 [10.5] years; 430 Hispanic or Latino [85%] 413 Spanish-speaking [82%]) participated. Preworkshop and postworkshop surveys (244 attendees) indicated significant improvements in self-reported knowledge, skills, and confidence (*t* test values ranging from −8.01 to −3.99). Additionally, qualitative data highlighted the workshops’ cultural relevance and the desire for more resources and extended engagement opportunities.

**Conclusions and Relevance:**

The findings of this study indicate that this CHW/Ps workforce training model and infrastructure can support the dissemination of trusted public health information during public health emergencies and beyond. Using this model, teams can adapt the content to their specific needs, and the resulting network of trained CHW/Ps can be mobilized to rapidly disseminate evidence-based information across medically underserved and other communities of interest.

## Key Points

**Question:**

Are culturally and linguistically relevant capacity-building trainings for the community health worker and *promotora* (CHW/P) workforce scalable and effective, and what are best practices for developing these trainings?

**Findings:**

In this mixed-methods study of 505 CHW/Ps in California, participants had significant increases from preworkshop to postworkshop survey in self-ratings of knowledge, skills, abilities, and confidence related to the learning objectives of the capacity-building program. Multimethod qualitative data from participant listening sessions indicated that participants found the training to be feasible, acceptable, and effective; best practices were highlighted for planning, implementation, and dissemination stages.

**Meaning:**

These findings suggest that this scalable CHW/P training model, codesigned in partnerships with CHW/Ps and other stakeholders, can be adapted for a variety of topics and leveraged to disseminate information and increase trustworthiness in health care, science, and medical institutions.

## Teaser

This mixed-methods study analyzes quantitative and qualitative data on the perceived effectiveness of a capacity-building training model for the community health worker and *promotora* (CHW/P) workforce in California.

## Introduction

Community health workers and *promotores* (CHW/Ps) are a crucial public health workforce, supporting health promotion, access, and navigation of health and social systems.^1,2,3,4^ They are trusted messengers who often share similar racial, ethnic, and sociocultural backgrounds with the communities they serve. The extant literature has highlighted the benefits of CHW/Ps in terms of return on investment^5,6^ and their effectiveness in improving various health outcomes.^4,7^ They represent a critical workforce for disseminating evidence-based information and overcoming medical and scientific misinformation and mistrust.

Recent policies, including the expansion of Medicaid coverage for CHW/P services,^8^ have increased demand for CHW/Ps^4^ and the need for capacity development for this workforce. While CHW/P certification programs have increased to meet the Medicaid CHW benefit requirements,^9,10^ with many states developing their own trainings,^11^ these programs are not without limitations. They tend to be offered exclusively in English despite data indicating that nearly one-half of the CHW/P workforce offers services in a language other than English, are offered in formats that may not be accessible, and often come at a cost to the CHW/Ps and/or their organizations.^12,13,14^ Moreover, current certification programs are often unable to cover topics in depth, do not address continuing education needs, or adapt to emerging needs and information.^15,16^ Addressing these gaps is hugely needed to support the CHW/P workforce long-term.

Funded by the National Institutes of Health Community Engagement Alliance and the California Alliance Against COVID-19 (hereafter, Alliance), a community-academic partnership and CHW/P community of practice,^17^ Promotoras con Stanford en Acción, was formed to address ongoing capacity building needs among CHW/Ps and promote the dissemination of evidence-based information in local communities. This community-academic partnership originally developed a series of virtual workshops to address the limitations of existing CHW/P training programs. These initial 11 workshops supported CHW/Ps in 3 counties in Northern California with over 3.1 million residents. The workshops included both single topics, such as understanding Medi-Cal benefits and expansion, as well as a series of 2 to 3 workshops on a single topic (eg, resource navigation or mental health first-aid).

The initial success inspired 5 sites within the Alliance—Stanford University; University of Southern California; University of California, Davis; University of California, Merced; and University of California, Irvine—to collaborate on expanding the workshops statewide. The community partnerships and advisory board also expanded to represent statewide geographies. This expanded advisory board designed and planned a 6-part workshop series centered on mental health.

We describe the process and best practices for codesigning and coimplementing an innovative, scalable, and culturally and linguistically relevant model of capacity-building workshops for CHW/Ps across California to better meet the needs of medically underserved communities. We present data on the feasibility, acceptability, and effectiveness of the statewide CHW/P workshops.

## Methods

This mixed-methods study employed a longitudinal evaluation to assess the feasibility, acceptability, effectiveness, and opportunities to improve and optimize the implementation of CHW/P capacity-building workshop model. We adhered to the Standards for Reporting Qualitative Research (SRQR) reporting guideline.^18^ The study was reviewed and approved by the Stanford University School of Medicine and UCLA institutional review boards. The institutional review boards determined that ripple effects mapping sessions were not considered human participant research because they were informing educucation curriculum content; therefore, this study was exempt from informed consent.

### Setting and Participants

Initial workshops occurred in Northern California Bay Area counties between June 2022 and August 2023, followed by statewide workshops from September 2023 to March 2024. Workshops took place monthly in the evening for 6 consecutive months. They were facilitated in Spanish, the language preferred by most of the CHW/Ps, with simultaneous English interpretation. Each workshop featured an evidence-based topic, presented by experts from both academic and community organizations, and included a CHW/P speaker. Data Collection

Demographic characteristics were self-reported by participants during registration. We evaluated workshops’ effectiveness, feasibility, and acceptability using 4 complementary methods. First, we conducted preworkshop and postworkshop surveys administered via REDCap with unique participant links. Surveys evaluated (1) overall learning objectives before and after completion of the 6-part series and (2) workshop-specific learning objectives for each workshop. Surveys also evaluated cultural and linguistic acceptability of the content, usefulness and uniqueness of the content and resources in strengthening their work in the community, and accessibility issues. Second, we collected qualitative data via chat comments during the workshops and responses to open-ended questions on evaluation surveys to assess engagement during the sessions, types of questions CHW/Ps asked, and suggestions for future topics.

Third, we facilitated 2 ripple effects mapping^19,20^ sessions, a participatory evaluation method, which draws from qualitative inquiry to assess multilevel intended and unexpected impacts (eg, for the CHW/Ps themselves, their organizations, and the community) of a program. Sessions lasted approximately 2 hours and involved peer interviews, group reporting around key highlights from the peer interviews, facilitator probing to establish sequence of events (eg, “What happened during the workshops that facilitated that outcome?”), and visual mapping of key outcomes using a concept mapping software, Xmind version 8 (Xmind Ltd).^21^ Sessions which were stratified by participants’ language preference, with one session held in English and one in Spanish. For recruitment, a list of potential participants, stratified by language preference and geographic region within the state (Southern, Central, and Northern California) was developed. Email invitations were then sent, indicating that we had limited slots available. Participants received a gift card for their participation.

Fourth, we hosted a virtual listening session with CHW/P workshop attendees. Drawing from focus group methodology, listening sessions are commonly used in participatory research to gather input on broad topics, encourage attendee participation, and encourage trust building.^22,23^ Utilizing Zoom (Zoom Communcations Inc) breakout groups, the listening session covered 5 topics including evaluation and feedback, desired future workshop topics, CHW/Ps networking opportunities and learning community, barriers and facilitators to resource navigation in their local communities, and workforce development needs. All prior participants received invitations to the listening session, which was held at the same time as the other workshops.

### Data Analysis

Quantitative data were analyzed using descriptive statistics, paired sample *t* tests to examine mean differences between preworkshop to postworkshop survey responses and χ^2^ tests to examine demographic differences. Qualitative data from ripple effects mapping and listening sessions were analyzed using thematic analysis^24^ and lightening report methods for rapid qualitative synthesis involving identifying key themes, suggestions for improvement, and future directions.^25^ Two independent research team members (P.R.E. and Y.M.M.) with formal training and prior experience in qualitative methods^26,27^ coded the data using a hybrid inductive and deductive coding approach.^28^ Deductive (theory or framework-based) codes included codes suggested by the lightening report method (eg, barriers, facilitators, and insights on the context and relevance of each topic),^25^ as well as codes based on the ripple effects mapping methodology, including unexpected outcomes or impacts and new or strengthened connections as a result of participation. Themes were later reviewed with the broader coauthor and investigative team, as well as with the community advisory board. However, these discussions tended to center on actionable steps from the data that could improve future workshop offerings, as well as how the various academic and community partners involved could bring findings to their organizations to inform their own programming.

Qualitative data from chat comments and the question and answer (Q&A) feature on Zoom during the workshops, and open-ended text from evaluation surveys were analyzed using rapid qualitative analysis methods,^25,29^ assisted by large-scale language model applications within generative AI. Recent research suggests that AI tools produce thematic analysis with considerable overlap to that produced by experienced qualitative researchers.^30^ We utilized the Stanford Medicine Secure GPT,^31^ a protected health information–compliant and secure platform for using AI in research, and followed emergent guidelines for AI-assisted thematic analysis.^30,32^ Transcripts with open-ended comments and questions were loaded into the Stanford Medicine Secure GPT with a description of the file (eg, details of the number of items and sections in the document) and prompts inquiring about a summary of the main themes identifiable in the document. Two research team members (Y.M.M. and P.R.E.) then reviewed the AI-generated themes for coherence and usefulness and compared findings with debriefing notes taken after each of the workshops. All data were deidentified before analysis. Excel version 16 (Microsoft), SPSS version 30.0 (IBM), NVivo qualitative software version 2020 (Limivero), and Xmind^21^—a mind mapping software—were used for analysis. A 2-sided *P* < .05 was considered significant.

## Results

The initial 11 workshops reached 392 CHW/Ps, while 6 statewide workshops reached 660 registrations. Of these, 505 CHW/Ps (mean [SD] age, 47.1 [10.5] years; 469 female [93%]) attended at least 1 workshop. CHW/Ps who attended the statewide workshops were predominantly Hispanic or Latino (430 CHW/Ps [85%]) and Spanish-speaking (413 CHW/Ps [82%]), and 315 CHW/Ps (62%) learned of the series through other CHW/Ps (Table 1). At registration, 307 CHW/Ps (61%) represented a community-based organization and resided in 243 different zip codes across California. Among all attendees, 305 (75%) attended 2 or more workshops, 305 (60%) attended 3 or more workshops, 232 (46%) attended 4 or more workshops, 136 (27%) attended 5 or more workshops, and 71 (14%) attended all 6workshops in the statewide series. Moreover, 197 CHW/Ps received a certificate of completion after attending, synchronously, 4 or more workshops within the statewide series and completing final evaluations. Except for being more likely to speak English and have received their education in the US, post hoc analysis revealed no other significant demographic differences among CHW/Ps who registered but did not attend any workshops (155 CHW/Ps) and those who attended at least 1 workshop (505 CHW/Ps) (eTable 1 in Supplement 1).

**Table 1.** Demographic Characteristics of Statewide Workshop Series Participants

Characteristics	Total sample, No. (%) (N = 505)^a^
Language at registration	
Spanish	413 (82)
English	92 (18)
Age, mean (SD), y	47.1 (10.5)
Gender	
Female	469 (93)
Male	28 (6)
Nonbinary	1 (<1)
Prefer not to answer	7 (1)
Race and ethnicity^b^	
American Indian or Alaska Native	2 (<1)
Asian or Asian American	8 (2)
Black or African American	1 (<1)
Hispanic or Latino	430 (85)
White	15 (3)
Multiracial^c^	27 (5)
Prefer not to answer	22 (4)
Education	
Less than high school	71 (14)
High school or GED	151 (30)
Some college	76 (15)
Trade, technical, or vocational school	49 (10)
Bachelor’s degree	100 (20)
Graduate school	29 (6)
Prefer not to answer	29 (6)
Education completed in the US	
Yes	199 (39)
No	281 (56)
Prefer not to answer	25 (5)
Region	
Northern California	182 (36)
Central California	28 (6)
Southern California	279 (55)
Outside of California^d^	16 (3.)
Met eligibility for certificate of completion	197 (39)
Workshop attendance^e^	
≥1 Workshops	505 (100)
≥2 Workshops	378 (75)
≥3 Workshops	305 (60)
≥4 Workshops	233 (46)
≥5 Workshops	136 (27)
6 Workshops	71 (14)
Affiliated with a community-based organization at time of registration	307 (61)
How did you learn about the workshops?^f^	
Academic partners	55 (11)
Other community health workers	315 (62)
Affiliated organization	154 (30)
Prefer to self-describe^g^	27 (5)

Abbreviation: GED, general educational development.

^a^ Includes participants who attended at least 1 workshop and excludes 155 participants who registered but did not attend any workshops.

^b^ Attendees self-identified race and ethnicity at registration.

^c^ Includes attendees who self-identified as Hispanic or Latino and selected another race and ethnicity category.

^d^ Includes community health workers or *promotores *who were invited by their partner organizations in California.

^e^ Not mutually exclusive categories.

^f^ Multiple choice question; participants were asked to select all that apply.

^g^ Describes learning of the workshop through listserv and social media advertisement.

Table 2 showcases lessons, best practices, and practical tips for planning, implementing, and disseminating similar capacity-building workshops. These lessons and practices are the result of iterative feedback from CHWs/Ps and community partners in our advisory boards, as well as from workshop attendees, academic partners, speakers, and staff who supported the implementation. A key factor in our success was selecting workshop topics based on requests from CHW/Ps during previous workshops and evaluations, as well as direct input from the advisory board and their network of community partners. Our infrastructure also facilitated ongoing engagement. For example, workshops were housed on a website with access to additional multilingual resources and information (long format with in-depth details, as well as simple infographics and 1-page flyers) to support CHW/Ps with community outreach efforts (eFigure 1 in Supplement 1). CHW/Ps could also access links to recordings of prior workshops (with audio in both English and Spanish), view opportunities for additional capacity-building training, and find information on future workshop dates, topics, and registration details. Workshops included ample time for live Q&A and activities to promote community building, networking, and audience engagement. Other factors influencing success included the use of a regular Zoom format, as opposed to webinars, which limit attendees’ ability to connect with each other and with the presenters.

**Table 2.** Planning, Implementation, and Dissemination Best Practices

Phase	Preworkshop	During workshop	Postworkshop
Planning and promotion	Convene CHW/Ps and community advisory board to identify workshop topics and speakers (ideally 2 speakers: 1 academic and 1 community partner to present diverse perspectives and promote community-academic partnerships). Provide one-on-one support for CHW/Ps interested in attending.^a^ Create promotional flyers describing workshop topics and introducing speakers, including date and time, registration link, and QR code. Registration is only required once throughout entire series. Send reminder emails 1 week prior and 1 day prior to or the day of the workshop to all registered attendees. Meet one-on-one with speakers to review format, audience, and other logistics. Request speakers develop bilingual slides—content in both English and Spanish. Send preliminary slides to interpreters at least 1 day before for preparation around key constructs.	Join meeting space 10 to 15 minutes in advance to conduct a tech-check (ie, PowerPoint, audio, screen sharing, and simultaneous interpretation) and convene with community and academic partners prior to starting the workshop. Enable Q&A function to monitor and filter questions during presentations. Designate interpreters prior to opening the Zoom room to attendees. Present slides with community norms, series overview, certificate opportunity, evaluation reminders from previous workshops, instructions for access to simultaneous interpretation, relevant trigger warnings, and recording reminders. Encourage attendees to introduce themselves and their organization (if applicable) in the chat to facilitate social connection with other CHW/Ps and hosts.	Promote next workshops at the end as a reminder for all attendees.
Implementation	Set Zoom meeting as a regular meeting (not webinar) to facilitate connection between CHW/Ps in attendance and facilitate discussion with speakers during Q&A. If needed, do voice-overs for any videos in the presentation that are only available in 1 language to make them linguistically accessible to CHW/Ps synchronously attending.	Give reminders of community norms and confidentiality before starting. Monitor chat for questions or comments shared during the workshops. Have designated staff for chat interactions (eg, adding key links, responding to technology questions, and encouraging adding questions to Q&A function). Allocate approximately 20 minutes for the Q&A section. Begin with 1-2 frequently asked questions from the chat or the Q&A function. Open the Zoom space for attendees to unmute to ask their questions directly. Remind attendees to keep questions relevant to overall audience and concise to allow for greater number of questions answered live.	Remind attendees of the website for later access and upcoming workshop recording. Thank speakers and interpreters for their collaboration and support participating in the workshop.
Evaluation	Create general series objectives and include in workshop registration to reassess at series completion to conduct overall pre- and postworkshop series analysis. Develop workshop-specific learning objectives to assess group knowledge prior to and after each workshop. In workshop reminders, send each registered CHW/P an individualized nontransferable survey link with workshop learning objectives to assess preworkshop knowledge and to facilitate pre- and postevaluation after the workshop.	Track attendance for postworkshop survey dissemination (only delivered to those registered and synchronously joining the workshop). Update certificate tracking documents as needed (eg, facilitating calculation of total workshops attended synchronously if this is a certificate requirement).	Send CHW/Ps a thank you email after attending the workshop and share personalized postworkshop evaluation. Debrief with team members and advisory board. If received, incorporate any feedback from speakers. Solicit feedback from presenters for iterative improvement of workshop processes. Provide one-on-one support for CHW/Ps with inquiries about the status of their evaluations, access to personalized evaluations, and progress in meeting certificate criteria.
Dissemination	NA	Record the workshop for later editing (eg, condensing to presentation and Q&A segments and editing audio in both English and Spanish if there were recording issues).	Publish edited recordings both in English and Spanish on website for accessibility and asynchronous use. Upload relevant resources shared during the workshop (eg, 1-page summaries, website links, hotlines)

Abbreviations: CHW/P, community health workers and *promotores*; NA, not applicable; Q&A, question and answer.

^a^ Support included but was not limited to assistance with registration, confirm language needs, and verifying completion.

Preworkshop and postworkshop surveys (244 participants) revealed significant increases in self-ratings for knowledge, skills, abilities, and confidence related to the learning objectives (*t* test values ranging from −8.01 to −3.99) (Table 3). While there were no significant differences in baseline self-reported knowledge by attendance (eTable 2 in Supplement 1), post hoc analyses indicated that participants who attended a greater number of sessions, commensurate with receipt of a certificate, were more likely to endorse significant changes in learning objectives (Table 4).

**Table 3.** Findings From Quantitative Statewide Workshop Series Evaluation

Question	Mean (SD)		Statistical test^a^	*P* value
	Preworkshop	Postworkshop		
Overall pre- and postlearning objectives (n = 244)^b^				
I feel confident in my understanding and ability to describe the mental health needs of my community.	4.0 (0.9)	4.3 (0.75)	−4.66	<.001
I know of available and free mental health assessments (eg, general, for anxiety, depression, stress, etc.) that I can use in my community.	3.6 (1.0)	4.1 (0.90)	−8.01	<.001
I feel confident in my ability to listen, speak, and communicate with diverse community members around mental health.	4.1 (0.9)	4.4 (0.78)	−4.35	<.001
I feel confident that I convey empathy and hope when discussing mental health topics with my community.	4.2 (0.9)	4.4 (0.75)	−3.99	<.001
I feel confident in my ability to connect community members with needed mental health resources.	4.0 (1.0)	4.4 (0.80)	−5.57	<.001
I know about local mental health resources and how to help members of my community navigate them.	3.7 (1.0)	4.2 (0.86)	−8.13	<.001
I feel confident in my ability to address mental health issues in my community.	3.8 (1.0)	4.3 (0.79)	−7.57	<.001
I have a network or group of individuals (community health workers, organizations) that I can reach out to for support or when I have questions about mental health topics.	3.7 (1.1)	4.2 (0.88)	−6.91	<.001
Acceptability across the statewide workshops, No (%) (n = 412)^c^^,^^d^				
I learned about a new resource during this workshop.	NA	393 (95)	NA	NA
Resources shared will improve my ability to work with my community.	NA	393 (95)	NA	NA
I feel confident I can apply what I learned in my daily work.	NA	397 (96)	NA	NA
I would recommend this workshop to other CHW/Ps outside of this network.	NA	395 (96)	NA	NA
Additional evaluation questions included in the final workshop, No. (%) (n = 179)^d^^,^^e^				
I have gained new skills because of these workshops.	NA	171 (96)	NA	NA
I am using skills and tools from the workshops in my daily work.	NA	172 (96)	NA	NA
I have learned about new resources available in my community as a result of attending these workshops.	NA	172 (96)	NA	NA
I feel comfortable sharing information from these workshops with other CHWs.	NA	170 (95)	NA	NA
I have made or strengthened connections with others as a result of attending these workshops.	NA	155 (87)	NA	NA
These workshops play an important role in advancing my work as a CHW.	NA	172 (96)	NA	NA

Abbreviations: CHW, community health worker; CHW/P, community health worker and *promotores*; NA, not appliable.

^a^ Statistical test: paired sample *t* test on SPSS.

^b^ Total number of CHWs who completed preassessment at registration and postassessment in the closing evaluation; this number does not entail completion of individual workshop evaluations.

^c^ Includes all participants who completed at least 1 evaluation.

^d^ Response options for all questions were (1) strongly disagree, (2) disagree, (3) neither agree nor disagree, (4) agree, and (5) strongly agree. The totals reflect those who endorsed agree or strongly agree. An agree or strongly agree response for any of the 5 workshops was counted once to indicate the total number of participants whom at any point in time endorsed the statement.

^e^ Total number of participants who completed evaluations after the listening sessions, which were held during the last workshop in the series.

**Table 4.** Quantitative Evaluation of Overall Learning Objectives From Statewide Workshop Series by Attendance Groups^a^

Questions^b^	Attended 1-3 workshops (n = 41)				Attended 4-6 workshops (n = 203)			
	Mean (SD)		Statistical test^c^	*P* value	Mean (SD)		Statistical test^c^	*P* value
	Preworkshop	Postworkshop			Preworkshop	Postworkshop		
I feel confident in my understanding and ability to describe the mental health needs of my community.	4.3 (0.8)	4.2 (0.8)	1.22	.23	4.0 (1.0)	4.4 (0.7)	−5.26	<.001
I know of available and free mental health assessments (eg, general, for anxiety, depression, stress, etc.) that I can use in my community.	3.6 (1.0)	4.1 (0.9)	−3.38	.002	3.6 (1.0)	4.1 (0.9)	−7.31	<.001
I feel confident in my ability to listen, speak, and communicate with diverse community members around mental health.	4.1 (0.9)	4.2 (0.9)	−0.84	.41	4.1 (0.9)	4.4 (0.8)	−4.36	<.001
I feel confident that I convey empathy and hope when discussing mental health topics with my community.	4.1 (0.9)	4.2 (0.9)	−0.19	.85	4.2 (0.9)	4.2 (0.7)	−4.19	<.001
I feel confident in my ability to connect community members with needed mental health resources.	4.2 (0.8)	4.4 (0.8)	−2.47	.02	4.0 (1.0)	4.4 (0.8)	−5.16	<.001
I know about local mental health resources and how to help members of my community navigate them.	3.8 (0.3)	4.2 (0.8)	−3.37	.002	3.7 (1.0)	4.2 (0.9)	−7.44	<.001
I feel confident in my ability to address mental health issues in my community.	3.9 (1.0)	4.2 (0.9)	−2.48	.02	3.8 (1.0)	4.3 (0.8)	−7.16	<.001
I have a network or group of individuals (community health workers, organizations) that I can reach out to for support or when I have questions about mental health topics.	3.9 (1.1)	4.1 (1.0)	−1.27	.21	3.7 (1.1)	4.3 (0.9)	−7.04	<.001

^a^ Includes 244 community health workers who completed preassessment at registration and postassessment in the closing evaluation.

^b^ Response options for all questions included (1) strongly disagree, (2) disagree, (3) neither agree nor disagree, (4) agree, and (5) strongly agree

^c^ Statistical test: paired-sample *t* test in SPSS.

Chat comments (1424 comments) and open-ended responses on evaluation surveys (1337 responses from 385 unique CHW/Ps) revealed cross-cutting themes across the workshops (Table 5), including positive feedback on the presentations and usefulness of the content and resources shared, including valuing how speakers integrated cultural issues and shared practical tips and resources. Participants expressed an understanding of social determinants of health as root causes of many of the issues being discussed and the need for systemic change. They also expressed a desire for additional resources in their own communities, as well as a desire for additional workshops, including additional time for each topic and further opportunities for interactions with speakers and with each other. Participants also utilized the chat and evaluations to express appreciation for the language access and culturally relevance of the content, while also suggesting additional ways to increase access to the materials (eg, having all handouts, resources, and slides offered in multiple languages). While feedback was overwhelmingly positive, on average, we received a mean (range) of 17 (16-31) chats or open-text evaluation comments per workshop with constructive criticisms, most often around technology and audio issues, confusion around where to find evaluation links, and a desire for more discussion time and fully bilingual slides.

**Table 5.** Mixed-Methods Evaluation Data

Overall mixed-method finding	Evidence	
	Quantitative	Qualitative
Finding 1: Increased comprehension of mental health (eg, prevention and identification) and ways to address it	Survey findings: Of 244 CHW/Ps, 225 (92%) were confident in their ability to understand and describe mental health needs, compared with 188 (77%) in the preworkshop series.^a^ Of the CHW/Ps, 225 (92%) were confident listening, speaking, and communicating with diverse community members around mental health needs compared with 195 (80%) in the preworkshop series.^a^ Of all CHW/Ps, 217 (89%) were confident addressing mental health needs in their communities compared with 168 (69%) in the preworkshop series.^a^	Open-ended evaluations: “The presenter used simple language and examples that helped me understand and differentiate depression, stress, and anxiety.” Evaluation workshop 3
		REM: “Better understanding of the community (eg, understanding that topics might be triggering for that community and now applying ground rules, breathing exercises, etc. to support.” English REM session
		Listening session: “It is very important for us as a community to have a broader understanding of the situations we can encounter…of how to address the topic…and support the person that needs help in that moment.” Evaluation breakout group
Finding 2: Greater understanding of navigation and mental health resources in the community	Survey findings: Of 244 CHW/Ps, 222 (91%) were confident in their ability to connect community members with mental health resources compared with 188 (77%) in the preworkshop series.^a^ Of all CHW/Ps, 213 (87%) knew about local mental health resources and navigation compared with 151 (62%) in the preworkshop series.^a^	Open-ended evaluations: “By patiently listening and directing people to the resources they need. Informing people so they can make informed decisions. Helping others feel empowered.” Evaluation workshop 6
		REM: “Learned more about how to connect people with resources.” Spanish REM session
		Listening session: “We also learned about 988, that is by area…We have contact with these organizations or groups that can help with a psychologist, psychiatrist, or something more specific. At the same time there are other organizations that have workshop for our Latino community in our language.” Resource navigation breakout group
Finding 3: Facilitation of connections to other CHW/Ps and organizations	Survey findings: Of 244 CHW/Ps, 210 (86%) endorsed having a network of other CHW/Ps and organizations to connect with for support or questions regarding mental health compared with 163 (67%) in the preworkshop series.^a^	Open-ended evaluations: “It made me really happy to see other colleagues connected and happy to learn. Thank you for the opportunity to access more education to continue helping others.” Final evaluation
		REM: “Connections to people attended from all over the state and other counties; contact information in the chat helped for others to make the connection and share safely.” English REM session
		Listening session: “We do have support networks…as is this one, when we have had a question about a service that we do not know about, we send chats and receive answers.” Networking breakout group
Finding 4: Culturally and linguistically accessible information	Survey findings: Of 412 CHW/Ps, 383 (93%) on average reported workshops improved their ability to work with their communities, and 387 (94%) on average reported feeling confident applying what was learned in workshops in their daily work.	Open-ended evaluations: “Thank you for the language justice it is very important for our Spanish-speaking community to have a voice in these spaces.” Evaluation workshop 1
		REM: “Stories and examples are useful for when she later speaks with community members and can use them.” Spanish REM session
		Listening session: “I’ve studies some of these topics in English but having it in Spanish was very useful. It gives us the right vocabulary to teach others and to be useful in our communities.” Workshop evaluation breakout group
Finding 5: Impact on day-to-day activities with the community	Workshop series objectives: Of 244 CHW/Ps, 230 (94%) were confident they could convey empathy and hope when discussing mental health topics with their community compared with 205 (84%) in the preworkshop series.^a^	Open-ended evaluation: “The skills and tools shared in these workshops have helped me have conversations regarding trauma and mental health with Latino families. I have practiced my motivational interviewing skills and been able to have conversations about managing stress, mental health, and practicing mindfulness.” Evaluation workshop 6
		REM: “I was able to take the information and facilitated a group that includes teens and 20-25 people in attendance. I’m doing it with a co-worker and bringing additional folks within [my] organization.” English REM session
		Listening session: “We, as community promoters, have a big role to play in supporting our communities, especially those without access to resources. These resources you’ve shared help us know where to refer them.” Resource navigation breakout group
Finding 6: Workshops’ acceptability	Series metrics: A total of 660 CHW/Ps registered for the series, 505 attended at least 1 workshop; on average, 318 CHW/Ps joined workshops synchronously, 378 (75%) attended 2 or more workshops, 232 (46%) attended 4 or more workshops, and 71 (14%) attended the entire series. A total of 197 CHW/Ps received a certificate after attending at least 4 workshops synchronously, completing respective evaluations, and a closing evaluation.	Open-ended evaluations: “I found the topics and sequence of the workshops interesting, fluid and clear as basic information to understand and to be able to take that information to the community...a way to educate the promoters...and develop [skills] and put it into practice in the community. In short, the format was good, it gave us all the opportunity to participate.” Evaluation workshop 6
		REM: “I was very excited and would make notes during the workshops. Very happy that I can help the community based on those notes.” English REM session
		Listening session: “It is an additional certificate…for promotoras learning here…it opens doors within the organization to promote more job contracts.” Workforce development breakout group

Abbreviations: CHW/Ps, community health workers and *promotores*; REM, ripple effects mapping.

^a^ Percentages calculated from CHW/Ps who endorsed agreed and strongly agree response options to overarching learning objectives at the start and end of the workshop series.

Ripple effects mapping sessions (26 CHW/Ps) revealed expected and unexpected outcomes of the workshops including increasing knowledge and skills, facilitating and deepening connections among the CHW/Ps, learning about resources in other counties or the state, and dissemination of workshops’ content via additional outreach and educational sessions hosted by the CHW/Ps in their local communities. Participants expressed a desire for additional workshops, longer workshops, and additional support in disseminating information in their respective communities (eFigure 2 in Supplement 1).

Listening sessions (280 CHW/Ps) further highlighted the perceived importance of these workshops in enhancing CHW/Ps’ knowledge and skills to better serve their communities. CHW/Ps saw the workshops as accessible and effective in increasing their understanding of mental health topics, prevention, and evidence-based coping strategies, and for enhancing their confidence in disseminating resources (Table 5). Participants also expressed the need for ongoing capacity building in other health and population-specific topics, given the challenges CHW/Ps face when working with community members with complex social and health needs.

## Discussion

Findings from our mixed-methods study using quantitative and qualitative analysis showcased the feasibility, acceptability, and effectiveness of our culturally and linguistically relevant capacity-building trainings for CHW/Ps. Our scalable model and implementation process, codesigned in partnerships with CHW/Ps and other stakeholders, can be adapted for a variety of topics applicable to the CHW/P workforce. It can also be leveraged for disseminating information and to increase trustworthiness in health care, science, and medical institutions. Our multimethod evaluation underscored positive impacts for the CHW/Ps, their organizations and networks, and the communities they serve, and also allowed us to learn about broader workforce challenges and needs.

Our findings contribute to the emerging literature on CHW/Ps’ workforce development and readiness,^33,34^ including recommendations by the World Health Organization.^35^ Prior studies have found that professional growth, including training opportunities, is an important factor for enhancing CHW/Ps’ performance and retention.^34,36^ Our training model addresses continuing education needs, including annual requirements of the Medicaid CHW benefit (minimum of 6 continuing education hours annually), and has the potential to train this workforce on specific topics relevant to public health and health care.

### Implementation Recommendations and Sustainability

Listening attentively to the needs and priorities of the CHW/Ps, particularly in the topic selection, building and maintaining trust, and fostering trustworthiness among the partners was fundamental for our ongoing success and popularity among the CHW/Ps attendees. Offering the workshops in Spanish was also often highlighted as a unique feature that made our workshops popular among CHW/Ps for receiving information in their preferred language, thus addressing a limitation of many other capacity-building efforts, which are offered primarily in English. Interactive features, such as chat, and regular Zoom meeting set up, and the ability to unmute to ask questions during the Q&A portions, were also emphasized by participants as allowing for engagement, networking, and enhancing the learning experience.

Once the workflow is in place, workshops can be carried out with minimal funding by leveraging in-kind resources, professional networks to find speakers, and existing telecommunication infrastructures from participating institutions and organizations. Funding may still be needed for interpretation and similar professional services. Our partnerships with 5 academic institutions also allowed for additional staff support and task distribution, with minimal burden on each of the organizations. By leveraging our respective networks, reach and dissemination across the state CHW/Ps networks required minimal maintenance. We built a robust registration list, which, in combination with word of mouth within the CHW/Ps networks, served as the main mechanism for promotion. Indeed, our most successful recruitment strategy was word of mouth among the CHW/Ps, which can add sustainability for teams looking to implement similar trainings and serve as proxy for training acceptability and satisfaction.

Notably, higher attendance was associated with significant improvements in self-reported knowledge gains, consistent with the existing literature on group-based and nontraditional learning approaches.^37,38^ Thus, efforts should be made to encourage attendance formally (eg, via certificates of completion) and informally (eg, engaging activities, removal of attendance barriers). Finally, built-in surveys and other forms of evaluation also allow for timely feedback from CHW/Ps on ongoing needs that serve to plan future workshop topics that address timely priorities in the community.

### Future Directions

This capacity-building model can be adapted for various topics, including specific health issues, populations, or geographic needs (eg, CHW/Ps in rural areas). The model can also be adapted and offered by nonacademic medical centers. Future efforts should assess the quantity and types of resources required to support CHWs/Ps in disseminating information within their communities. While our evaluation captured some efforts by the CHWs/Ps to use the materials to develop their own workshops for community members and to adapt and disseminate the information in their local communities, additional systematic efforts and resources are likely needed to objectively assess use and to optimize outreach and dissemination in local communities. Moreover, given the high preworkshop mean scores, additional engagement of end users and psychometric work are recommended to refine the survey measure.

### Limitations

This work is not without limitations. Our workshops were facilitated in Spanish, which may limit the generalizability to other populations—particularly states with different languages and CHW/P demographics. While we offered synchronous interpretation to English, multilingual slides, and English audio for recordings, the workshops attracted a primarily Spanish-speaking or bilingual CHW/P population. However, this was also a strength, meeting a need for training in other languages preferred by the CHW/Ps. Additionally, the lack of randomization and the potential for self-selection bias limit causal inference about the workshops’ true effectiveness. Further, due to resource limitations, we were unable to follow participants long-term to assess the sustainability of the learnings or the objective use of materials and learnings in their respective organizations and communities.

## Conclusions

In this mixed-methods study using a quantitative and qualitative approach, we described the process and best practices for codesigning and coimplementing a scalable, culturally and linguistically relevant capacity-building model for CHW/Ps. As the CHW/P workforce continues to develop, there is a growing need for continuing education, as well as more in-depth trainings that can address specific topics and emerging needs. Through this statewide partnership, we were able to codesign and coimplement a series of evidence-based and scalable capacity-building workshops. This promising and scalable model, its associated infrastructure, and the resulting network of activated and trained CHW/Ps can serve as an avenue for rapid dissemination of evidence-based information and can become a trusted resource during public health emergencies.

## Notes

Supplement 1. eTable 1. Demographic characteristics of participants by attendance eFigure 1. Annotated website infrastructure for the community health worker/promotores workshops eTable 2. Comparison of baseline self-reported knowledge by attendance eFigure 2. Key findings from ripple effects mapping session

Supplement 2. Data Sharing Statement
