# Supplementary material for: Strengthening Community Health Worker and Promotora Workforce Capacity
Source: JAMA Netw Open. 2026 Apr 10;9(4):e266037. doi: 10.1001/jamanetworkopen.2026.6037 (PMC13069451; doi:10.1001/jamanetworkopen.2026.6037)
Supplement: Supplement 1. — eTable 1. Demographic characteristics of participants by attendance eFigure 1. Annotated website infrastructure for the community health worker/promotores workshops eTable 2. Comparison of baseline self-reported knowledge by attendance eFigure 2. Key findings from ripple effects mapping session [file jamanetwopen-e266037-s001.pdf]

Supplemental Online Content

Rodriguez Espinosa P, Martinez Mulet Y, Wolfe N, et al. Strengthening Community health worker and promotora workforce capacity. *JAMA Netw. Open*. 2026;9(4):e266037. doi:10.1001/jamanetworkopen.2026.6037

eTable 1. Demographic characteristics of participants by attendance
eFigure 1. Annotated website infrastructure for the community health worker/promotores workshops
eTable 2. Comparison of baseline self-reported knowledge by attendance
eFigure 2. Key findings from ripple effects mapping session

This supplemental material has been provided by the authors to give readers additional information about their work.

© 2026 Rodriguez Espinosa P et al. *JAMA Network Open*.

eTable 1. Demographic characteristics of participants by attendance

Characteristics	Attended 0 Workshops (n=155)	Attended 1-3 Workshops (n=273)	Attended 4-6 Workshops (n=232)	Statistical test (*P* value)
Language at registration				17.682 (<0.001)[a]
Spanish	102 (66%)	224 (82%)	189 (81%)	
English	53 (34%)	49 (18%)	43 (19%)	
Age, mean (SD)	44.5 (12.8)	47.2 (10.7)	47.0 (10.2)	3.062 (.05)[b]
Gender				3.324 (.81)[c]
Female	141 (91%)	254 (93%)	215 (93%)	
Male	12 (7.7%)	14 (5.1%)	14 (6.0%)	
Non-binary	1 (0.6%)	1 (0.4%)	0 (0%)	
Prefer not to answer	1 (0.6%)	4 (1.5%)	3 (1.3%)	
Race/Ethnicity				18.181 (0.06)[d]
American Indian or Alaska Native	0 (0%)	0 (0%)	2 (0.9%)	
Asian or Asian American	5 (3.2%)	1 (0.4%)	7 (3%)	
Black or African American	0 (0%)	0 (0%)	1 (0.4%)	
Hispanic or Latino	129 (83%)	240 (88%)	190 (82%)	
Multiracial	6 (3.9%)	12 (4.4%)	15 (6.5%)	
White	5 (3.2%)	11 (4%)	4 (1.7%)	
Prefer not to answer	10 (6.5%)	9 (3.3%)	13 (5.6%)	
Education				9.970 (.62)[a]
Less than high school	19 (12%)	37 (14%)	34 (15%)	
High school or GED	31 (20%)	80 (29%)	71 (31%)	
Some college	25 (16%)	45 (16%)	31 (13%)	
Trade/technical/vocational school	22 (14%)	25 (9.2%)	24 (10%)	
Bachelor's degree	36 (23%)	54 (20%)	46 (20%)	
Graduate school	13 (8.4%)	16 (5.9%)	13 (5.6%)	
Prefer not to answer	9 (5.8%)	16 (5.9%)	13 (5.6%)	
Education completed in the US				19.224 (<.001)[a]
Yes	88 (57%)	115 (42%)	84 (36%)	
No	58 (38%)	148 (54%)	133 (57%)	
Prefer not to answer	9 (5.8%)	10 (3.7%)	15 (6.5%)	
Region				
Northern CA	63 (41%)	95 (35%)	87 (38%)	

© 2026 Rodriguez Espinosa P et al. *JAMA Network Open*.

Central CA	4 (2.6%)	12 (4.4%)	16 (6.9%)	9.843 (.13)[a]
Southern CA	78 (50%)	155 (57%)	124 (53%)	
Outside of CA[e]	10 (6.5%)	11 (4.0%)	5 (2.2%)	
Community-based organization				
Affiliated at time of registration	81 (52%)	167 (61%)	140 (60%)	3.925 (.42)[a]

Notes. N=660 includes all complete registration; demographics are stratified by workshops attended. N=155 indicates registered individuals who attended 0 workshops. N=273 indicates registered individuals who attended 1-3 workshops. N=232 indicates registered people who attended 4-6 workshops.

[a]Statistical test: Chi-Squared test of independence

[b]Statistical test: One-way ANOVA.

[c]Fisher-Freeman-Halton Exact Test given 20% or more of cells had expected count less than 5.

[d]Monte Carlo simulation conducted in SPSS to obtain Fisher-Freeman-Halton Exact Test. 95% CI [0.056, 0.069] with $N = 10000$ sample.

[e]Includes CHW/Ps outside of CA who were invited by their partner organizations in CA.

© 2026 Rodriguez Espinosa P et al. *JAMA Network Open*.

eFigure 1. Annotated website infrastructure for the Community Health Worker/Promotores Workshops

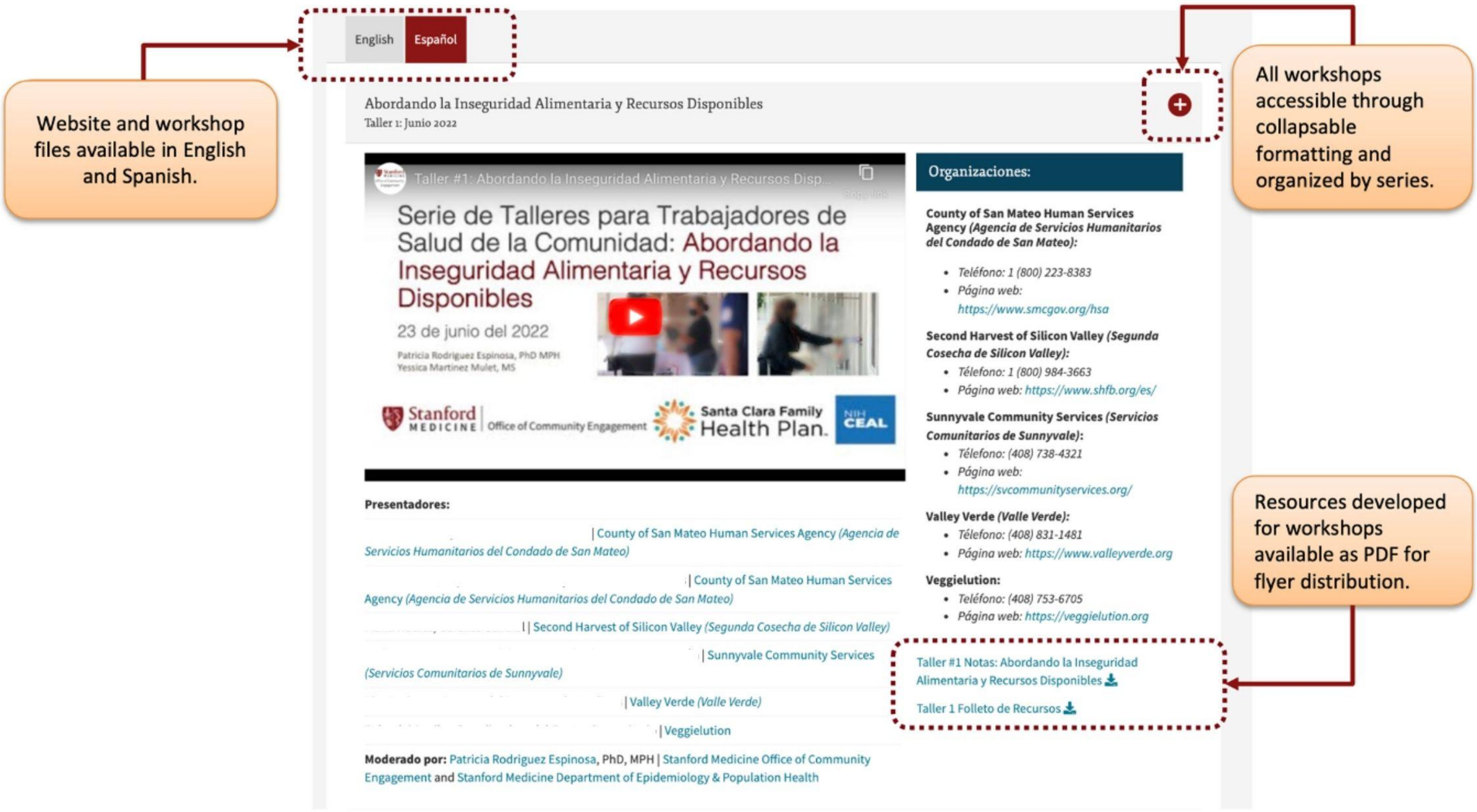

Notes. Speaker names redacted.

© 2026 Rodriguez Espinosa P et al. *JAMA Network Open*.

eTable 2. Comparison of baseline self-reported knowledge by attendance.

Pre-workshop Questions	Attended 0 Workshops (n=155)	Attended 1-3 Workshops (n=273)	Attended 4-6 Workshops (n=232)	***Statistical Test (P value)***[a]
	Mean (SD)	Mean (SD)	Mean (SD)	
I feel confident in my understanding and ability to describe the mental health needs of my community.	3.90 (1.1)	3.96 (.96)	4.0 (.96)	.41 (.66)
I know of available and free mental health assessments (e.g., general, for anxiety, depression, stress, etc.) that I can use in my community.	3.55 (1.18)	3.45 (1.04)	3.52 (1.01)	.55 (.58)
I feel confident in my ability to listen, speak, and communicate with diverse community members around mental health.	4.08 (1.06)	3.94 (.97)	4.09 (.85)	1.81 (.17)
I feel confident that I convey empathy and hope when discussing mental health topics with my community.	4.17 (.95)	4.05 (.91)	4.18 (.87)	1.47 (.23)
I feel confident in my ability to connect community members with needed mental health resources.	3.99 (1.01)	3.90 (.97)	3.98 (.96)	.53 (.59)
I know about local mental health resources and how to help members of my community navigate them.	3.61 (1.09)	3.60 (.96)	3.67 (.92)	.29 (.75)
I feel confident in my ability to address mental health issues in my community.	3.75 (1.04)	3.71 (1.02)	3.81 (.94)	.63 (.53)
I have a network or group of individuals (community health workers, organizations) that I can reach out to for support or when I have questions about mental health topics.	3.72 (1.15)	3.67 (1.09)	3.72 (1.04)	.17 (.85)

Notes. Response options for all questions: 1 = strongly disagree, 2 = disagree, 3 = neither agree nor disagree, 4 = agree, 5 = strongly agree

N=660 includes all who registered for the workshop series regardless of actual attendance.

[a]Statistical test: One-Way ANOVA; F and P-Values reported.

© 2026 Rodriguez Espinosa P et al. *JAMA Network Open*.

eFigure 2. Key findings from Ripple Effects Mapping session

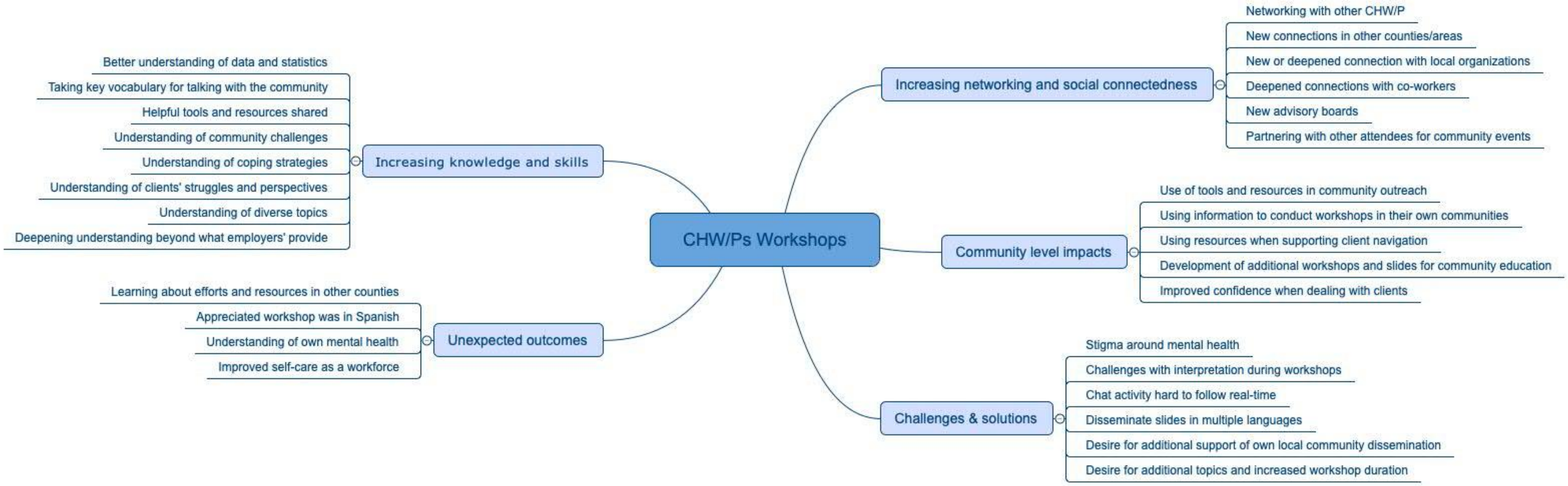

Note. Combined simplified map including findings from English and Spanish-speaking session.

© 2026 Rodriguez Espinosa P et al. *JAMA Network Open*.
