# Supplementary material for: Strengthening Community Health Worker and Promotora Workforce Capacity
Source: JAMA Netw Open. 2026 Apr 10;9(4):e266037. doi: 10.1001/jamanetworkopen.2026.6037 (PMC13069451; doi:10.1001/jamanetworkopen.2026.6037)
Supplement: Supplement 2. — Data Sharing Statement [file jamanetwopen-e266037-s002.pdf]

Data Sharing Statement

Rodriguez Espinosa. Strengthening Community Health Worker and Promotora Workforce Capacity. *JAMA Netw Open*. Published April 10, 2026.
doi:10.1001/jamanetworkopen.2026.6037

Data
Data available: No

Additional Information
Explanation for why data not available: Data will be available upon request.
